# Cross-Segmental Transfer Effects of Lower Limb Cross-Training Priming on Upper Extremity Functional Recovery in Pediatric Unilateral Cerebral Palsy: A Randomized Controlled Trial

**DOI:** 10.3390/children13060731

**Published:** 2026-05-25

**Authors:** Tamer M. El-Saeed, Mohammed F. Elbanna, Ahmed M. Aboeleneen, Afnan M. Alkhateeb, Afnan S. Gmmash, Ohud A. Sabir, Abeer S. Jamal, Marwa M. I. Ismaeel

**Affiliations:** 1 Department of Physical Therapy, Faculty of Medical Rehabilitation Sciences, King Abdulaziz University, Jeddah 22252, Saudi Arabia; 2 Department of Physical Therapy for Pediatrics, Faculty of Physical Therapy, Cairo University, Giza 12611, Egypt; 3 Department of Basic Science, Faculty of Physical Therapy, Cairo University, Giza 12611, Egypt

**Keywords:** unilateral cerebral palsy, motor priming, cross-training, upper limb rehabilitation

## Abstract

**Background:** Upper extremity deficits in unilateral cerebral palsy (UCP) severely restrict daily autonomy. Although movement-based priming is known to stimulate neuroplasticity, the distal transfer of lower extremity (LE) training to augment paretic upper limb (UL) function remains largely uninvestigated. This randomized controlled trial evaluated whether a 6-week LE cross-training (CT) priming regimen enhances UL functional restoration in pediatric UCP. **Methods:** Thirty-six children (6–8 years) were randomized to a conventional physical therapy cohort (*n* = 18) or an experimental CT cohort (*n* = 18). The CT group performed high-resistance contractions utilizing the non-paretic LE immediately preceding standard therapy. Blinded evaluations quantified Handgrip Strength (HGS) via dynamometry, grasping proficiency via the Peabody Developmental Motor Scales (PDMS-2), and gross dexterity via the Box and Block Test (BBT) pre and post intervention. **Results:** Analysis indicated a robust, near-significant between-group effect (Wilks’ Λ = 0.775, *p* = 0.057). While both cohorts achieved substantial internal improvements, the CT participants displayed superior developmental trajectories across all domains, notably in grasping age equivalence (34.28 ± 6.33 vs. 25.78 ± 3.26 months) and HGS (3.89 ± 0.79 vs. 3.03 ± 0.53 kg). **Conclusions:** LE cross-training priming may be a feasible adjunct, but it did not demonstrate statistically significant additional UL benefit versus standard rehabilitation in this sample. Therefore, these results should be interpreted as exploratory and hypothesis-generating. This potential cross-segmental transfer may theoretically operate via interhemispheric facilitation, warranting further investigation in larger, adequately powered trials.

## 1. Introduction

Unilateral cerebral palsy (UCP) constitutes a significant subgroup within the broader spectrum of cerebral palsy (CP), characterized by motor impairments predominantly affecting one side of the body [1,2,3]. These impairments stem from a non-progressive lesion or disturbance occurring in the contralateral developing cerebral hemisphere [4,5]. Etiology is often linked to focal or asymmetric neurological events such as perinatal stroke, specific congenital brain malformations, or localized injury from factors like hemorrhage or infection [6,7]. While UCP is defined by its unilateral motor presentation, the clinical picture frequently involves a complex array of neurological deficits extending beyond the most affected limbs, impacting overall function and participation [8,9].

Children diagnosed with UCP commonly exhibit muscle weakness, alterations in muscle tone (typically spasticity), reduced joint mobility, difficulties with selective motor control, and often associated sensory processing deficits on their affected side [10,11]. Although lower limb dysfunction often dominates clinical attention due to its direct impact on gait and mobility, impairments of the affected upper limb are also highly prevalent and functionally consequential. Difficulties with reach, grasp, release, and bimanual coordination can significantly impede performance in activities of daily living, academic tasks, play, and vocational pursuits, potentially leading to learned non-use and further limiting functional independence and participation over the lifespan [12,13,14].

Consequently, comprehensive physical rehabilitation programs for children with UCP typically adopt a multifaceted approach. Standard interventions often include neurodevelopmental treatment (NDT) techniques, task-specific training, muscle strengthening exercises, passive and active stretching, and sometimes orthotic management, addressing impairments and functional goals across both upper and lower limbs [15,16,17]. In recent years, adjunct therapeutic strategies designed to leverage the principles of neuroplasticity, the brain’s inherent capacity for adaptive reorganization, have gained prominence in the field of neurorehabilitation. Movement-based priming represents one category of interventions [18,19]. Priming involves engaging in specific sensorimotor activities immediately prior to functional training or skill acquisition practice, with the theoretical aim of modulating neural circuits to create a state more receptive to motor learning and therapeutic benefit [20,21].

Cross-training (CT) is a specific form of unilateral movement-based priming grounded in the well-documented phenomenon of cross-education [22,23]. Cross-education refers to the transfer of motor performance gains, particularly strength, from a unilaterally trained limb to the homologous muscles of the contralateral, untrained limb. This effect is believed to be mediated by complex neurophysiological adaptations occurring primarily within the central nervous system. Proposed mechanisms include alterations in corticospinal excitability, modulation of inhibitory and excitatory pathways between the cerebral hemispheres (interhemispheric inhibition), adaptations within spinal cord networks, and potential contributions from shared neural circuits involved in motor planning and execution [24,25]. Many children with UCP demonstrate atypical corticospinal organization, often retaining persistent ipsilateral projections from the non-lesioned hemisphere due to early developmental reorganization. This unique neuroanatomical anomaly suggests that unilateral lower-limb training may engage shared or bilateral motor networks differently than in neurologically typical individuals. While cross-education is established for homologous muscles in adults, pediatric evidence remains scarce [26,27].

El-Saeed et al. suggested that strengthening-based CT priming protocols targeting the less-affected lower limb can be effective in improving motor function in the affected lower limb of children with UCP, likely through these cross-education mechanisms. Such findings support the potential of CT priming as a valuable component within a comprehensive rehabilitation plan focused on enhancing gait and mobility [23]. However, given the interconnectedness of the sensorimotor system and the frequent co-occurrence of upper and lower limb impairments in UCP, an important and largely unexplored question arises: Could an intensive priming intervention focused exclusively on the lower limb induce secondary or “carry-over” effects on the function of the contralaterally affected upper limb?

Several theoretical pathways could potentially mediate such cross-segmental transfer effects. Firstly, the neural adaptations induced by unilateral LL priming might not be strictly localized to LL representations in the sensorimotor cortex. Potential widespread changes in hemispheric excitability, modulation of interhemispheric balance, or activation of shared postural control networks could conceivably influence neural circuits controlling the upper limb within the affected hemisphere [28,29]. Secondly, improvements in lower limb function, dynamic balance, and overall postural stability achieved through the LL intervention could provide a more stable mechanical foundation or “proximal stability,” indirectly enabling more effective control and use of the affected upper limb during tasks requiring postural support or inter-limb coordination [30,31]. Thirdly, enhanced mobility or reduced effort during walking might liberate attentional resources, allowing for greater focus on, and potentially improved performance of, concurrent or subsequent upper limb activities [32]. While limb-to-limb transfer between homologous muscles is a documented motor learning phenomenon, the concept of heterologous, cross-segmental transfer (from lower to upper limbs) in a pediatric neurological population remains highly speculative. Consequently, the mechanisms proposed herein are strictly hypothetical. This study aims to address this research gap by providing exploratory data on the feasibility of this cross-segmental approach.

Therefore, the present study was designed to investigate the potential effects of a 6-week strengthening-based CT priming protocol, applied to the less-affected lower limb and added to a standard physical rehabilitation program, on the functional status of the affected upper limb in children aged 6–8 years with UCP. The primary objective was to compare changes in affected handgrip strength (HGS) and grasping skills, as measured by the Peabody Developmental Motor Scales–Fine Motor (PDMS-FM) Grasping subtest, between children receiving the combined LL CT priming and standard rehabilitation versus those receiving standard rehabilitation alone. We hypothesized that the group receiving the adjunct LL CT priming intervention would demonstrate significantly greater improvements in these secondary upper limb outcome measures compared to the control group receiving only standard rehabilitation.

## 2. Materials and Methods

### 2.1. Design Overview

The architectural framework of this investigation was established as a prospective, two-arm, parallel-group randomized controlled trial. The primary objective was to interrogate the clinical impact of an adjunct lower limb (LL) cross-training (CT) priming regimen on the restorative functional outcomes of the upper extremity (UL) in a cohort of children diagnosed with unilateral cerebral palsy (UCP). To ensure the highest level of bioethical transparency, the trial was conducted in strict conformity with the ethical standards of the 1964 Declaration of Helsinki and its subsequent amendments.

Formal administrative and ethical ratification were provided by the Institutional Review Board (IRB) of the Faculty of Physical Therapy, Cairo University, Egypt (Protocol No: P.T.REC/012/006149). In alignment with international transparency mandates for clinical research, the study was prospectively archived in the ClinicalTrials.gov registry (Trial Identifier: NCT07525752). Prior to any enrollment or investigative procedures, comprehensive verbal briefings were conducted, and formal written informed consent was secured from the legal guardians of all participants to ensure voluntary and informed inclusion.

### 2.2. Participants

Children with UCP were recruited from local pediatric rehabilitation centers and outpatient clinics between October 2025 and March 2026 to participate in this study. Eligibility screening identified children meeting the following inclusion criteria: (1) confirmed clinical diagnosis of spastic UCP [4]; (2) age between six and eight years at enrollment [33]; (3) Modified Ashworth Scale (MAS) grade for affected lower limb spasticity ranging from 1 to 1+ [34]; (4) ability to stand and ambulate independently without assistive devices (routine ankle-foot orthosis use was permitted); (5) ability to comprehend and follow simple verbal instructions necessary for testing and intervention participation. Exclusion criteria were presence of fixed musculoskeletal deformities in upper or lower limbs, significant cognitive or perceptual impairments interfering with participation, uncorrected visual or auditory deficits, comorbid conditions contraindicating participation (e.g., uncontrolled epilepsy, severe behavioral disorders), current use of systemic muscle relaxants or neurolytic agents, history of botulinum toxin injections to upper or lower limbs within the preceding six months, and prior orthopedic surgery on upper or lower limbs.

#### 2.2.1. Sample Size Calculation

To ensure the statistical sensitivity and inferential robustness of the trial, an a priori power analysis was performed using G*Power software (version 3.1.9.4; Heinrich Heine University, Düsseldorf, Germany). The calculation was specifically structured to identify clinically meaningful differences in the primary upper-limb functional outcomes between the investigative cohorts.

Drawing from established clinical literature in pediatric neurorehabilitation, an anticipated medium-to-large effect magnitude (Cohen’s d ≈ 0.80) was utilized for the calculation. With the Type I error rate (alpha) established at α=0.05 and a target statistical power of 80% (1−β=0.80) for a multivariate analysis framework, the analysis determined that a minimum of 18 participants per group was required. Consequently, a total cohort of N=36 children was targeted for recruitment to satisfy these statistical parameters, thereby avoiding attrition, mitigating the risk of Type II errors and ensuring a high level of confidence in the subsequent inter-group comparisons.

#### 2.2.2. Randomization and Blinding

Subsequent to the baseline diagnostic evaluations and the verification of eligibility, participants were stochastically partitioned in a 1:1 allocation parity into either the conventional physical therapy (PT) control group or the experimental cross-training (CT) priming group. To ensure prognostic balancing and mitigate the confounding influence of muscle tone severity, a stratified randomization strategy—predicated on the baseline Modified Ashworth Scale (MAS) scores—was executed via a computer-generated random sequence [35].

Stringent allocation concealment was maintained through the utilization of a Sequentially Numbered, Sealed, Opaque Envelope (SNOSE) protocol. These envelopes were managed by an independent researcher and were only unsealed following the completion of all enrollment procedures and initial functional assessments. Regarding methodological masking, the senior physical therapist responsible for the longitudinal acquisition of the upper and lower limb metrics remained strictly blinded to the group assignments throughout the trial’s duration. However, given the overt physical nature of the rehabilitative interventions, the masking of the treating therapists, participants, and their respective caregivers was not feasible—a recognized methodological constraint in clinical physiotherapy research [36].

### 2.3. Outcome Measures

Assessments were performed at baseline (pre-intervention) and immediately following the 6-week intervention period (post-intervention) by the blinded outcome assessor.

#### 2.3.1. Affected Handgrip Strength

Maximal isometric grip strength of the affected hand was quantified using a Jamar^®^ Plus+ digital handheld dynamometer (Patterson Medical, Cedarburg, WI, USA), capable of measuring force up to 90 kg and calibrated prior to use. Standardized positioning was strictly adhered to for all assessments to ensure reliability. Each child was seated upright in an adjustable-height chair providing appropriate back support, with their feet resting flat on the floor and both hips and knees maintained at approximately 90° flexion. The tested upper limb was carefully positioned according to established guidelines: the shoulder remained adducted and neutrally rotated alongside the trunk, the elbow was stabilized at 90° flexion, the forearm was held in a neutral (mid-prone/supine) orientation, and the wrist was maintained in slight extension (approximately 15°). Following familiarization with the device and standardized verbal encouragement to elicit maximal effort, the child was instructed to squeeze the dynamometer handle as forcefully as possible for several seconds before releasing it. This procedure was repeated three times for the affected hand, with a minimum rest period of 30 s enforced between trials to mitigate fatigue effects. The mean force value derived from these three maximal voluntary contractions, recorded precisely in kilograms (kg), was utilized for subsequent data analysis [37].

#### 2.3.2. Grasping Skills

Functional grasping abilities were formally evaluated using the Grasping subtest of the Peabody Developmental Motor Scales-Second Edition, Fine Motor scale (PDMS-2 FM), a widely recognized standardized assessment tool for evaluating fine motor development in young children. Administration of the subtest adhered strictly to the standardized protocol detailed within the official PDMS-2 manual to ensure procedural fidelity. Each child was assessed while comfortably seated at an appropriately sized table, positioned such that their feet could rest securely flat on the floor. The testing environment allowed the trained evaluator to sit either opposite or adjacent to the child as needed for optimal task presentation and observation. The Grasping subtest required the child to attempt a series of age-appropriate items involving various prehension patterns and object manipulation skills (e.g., picking up pellets, buttoning, holding a writing utensil). Performance on each task item was meticulously scored according to the specific criteria outlined in the PDMS-2 manual, yielding a subtest raw score. This raw score was converted into an age-equivalent score (expressed in months). Age-equivalent scores were utilized to provide a clinically interpretable metric of developmental “catch-up,” though we acknowledge their limitations regarding interval precision across varying ages. The PDMS-2 demonstrates excellent test–retest and inter-rater reliability (ICC ≥ 0.90) in pediatric neurodevelopmental cohorts [38,39].

#### 2.3.3. Manual Dexterity

Gross manual dexterity was evaluated using the standardized Box and Block Test (BBT). This widely utilized assessment involves a specific wooden box divided into two compartments by a central partition, along with 150 one-inch wooden cubes.

For administration, the box was placed at the participant’s midline on a table of appropriate height, with all blocks initially located in the compartment contralateral to the hand being tested. Following standardized instructions and a brief familiarization/practice trial (typically 15 s), the child was instructed to transfer as many blocks as possible, one at a time, over the central partition into the opposite compartment using only the specified hand within a strict 60 s time limit. Emphasis was placed on transporting only one block per attempt and ensuring fingers crossed the partition during transfer; blocks dropped outside the box were not counted unless retrieved solely with the tested hand. The test was administered separately for the affected hand. The score recorded for each hand was the total number of blocks successfully relocated to the designated compartment within the one-minute period [40].

The BBT has demonstrated good to excellent test–retest and inter-rater reliability in various populations, including studies involving children and individuals with neurological conditions, making it a consistent measure for assessing changes in gross manual dexterity [41].

### 2.4. Interventions

Participants in both groups received interventions five times per week for six consecutive weeks, delivered by experienced pediatric physical therapists. Total session time was equivalent between groups.

#### 2.4.1. PT Group (Passive Movement + Standard Rehabilitation)

Participants (n = 18) received a 15 min passive therapist-administered movement of the less affected limb, matched for limb, range of motion, body position, and total time followed by a 60 min multifaceted intervention divided into two discrete blocks to address the systemic nature of Unilateral Cerebral Palsy:

Upper Limb Functional Training (30 min): To address the primary outcome measures of this study, a Goal-Directed Task Training (GDTT) approach was implemented. This evidence-based protocol focused on the “Activity” and “Participation” domains of the ICF. Exercises targeted the paretic limb’s selective motor control, specifically focusing on reach-to-grasp patterns, bimanual coordination, and manual dexterity. Activities were individualized based on the child’s “meaningful goals” (e.g., handling cutlery, buttoning clothing, or manipulating school supplies). Therapeutic putty was used for intrinsic hand muscle strengthening, and repetitive task practice was employed to improve the quality of the “stabilizer–activator” relationship between the two hands [42].

Lower Limb and Axial Segment (30 min): This block focused on enhancing postural stability and locomotor efficiency. It incorporated neurodevelopmental treatment (NDT) principles to modulate muscle tone, individualized stretching of the triceps surae and hamstrings to maintain range of motion, and functional weight-bearing exercises. Task-specific gait training was utilized to improve symmetry and balance during ambulation.

#### 2.4.2. CT Group (Priming + Standard Rehabilitation)

**Figure 1:**
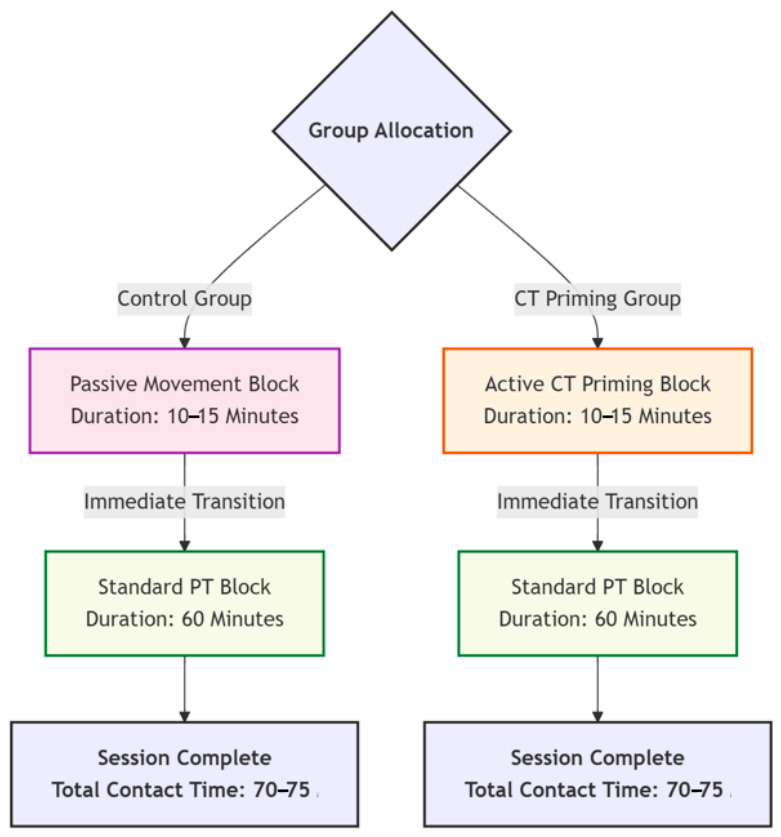
Schematic Timeline.

Participants (n = 18) assigned to the experimental cohort underwent a structured, exercise-mediated priming regimen targeting the less-affected lower extremity to catalyze neural remodeling. Administered by a senior pediatric physical therapist, this 10–15 min preparatory session was systematically scheduled to precede the standardized rehabilitation program. It is important to note that the interventions were time-matched [43,44]. The precise sequence of activities is illustrated in (Figure 1).

- Biomechanical and Contractile Specifics: The priming protocol targeted the primary muscle groups of the non-paretic limb, specifically the knee flexors, knee extensors, ankle dorsiflexors, and plantar flexors. Each exercise sequence integrated into a triphasic contraction pattern (comprising isometric, concentric, and eccentric phases) with each phase maintained for a strict duration of two seconds to ensure high-quality motor unit recruitment [45].
- Individualized Titration and Intensity: Training loads were tailored to each participant’s functional capacity based on a priori assessment of optimal resistance and repetition thresholds. To ensure a standardized physiological stressor, training intensity was calibrated to a moderate perceived exertion level (Borg CR-10: 4–6). Given the fluctuating muscle tone in UCP, formal 1RM testing was unfeasible. Instead, resistance was applied using pediatric elastic bands and manual therapist resistance, dynamically adjusted by the treating therapist during each session to maintain the target exertion level for 10-repetition sets [46].
- Volume and Recovery Parameters: The total training volume was limited to 30 repetitions per muscle group, subdivided into three discrete sets of 10 repetitions. To prevent peripheral fatigue from confounding the subsequent rehabilitation session, standardized recovery intervals of 2–3 min were enforced between sets.
- Preparatory and Recovery Phases: Every therapeutic session was initiated with a standardized 5 min pre-training conditioning phase, designed to elevate core body temperature and optimize neuromuscular readiness. This preliminary block comprised three minutes of sub-maximal aerobic engagement—such as slow-paced ambulation or unloaded cycle ergometry—followed by a two-minute sequence of rhythmic dynamic mobilization. These activities specifically included marching in place and controlled partial squats, aimed at enhancing multi-joint proprioceptive feedback and joint lubrication.

Following the completion of the primary intervention, a 5 min post-training recovery block was executed to facilitate systemic homeostatic restoration and minimize peripheral muscle soreness. This terminal phase consisted of three minutes of light aerobic cooling, culminating in two minutes of sustained static elongation. These stretches meticulously targeted the gastrocnemius-soleus complex (triceps surae), hamstrings, and quadriceps musculature, with the objective of promoting muscular relaxation and maintaining tissue extensibility after the high-intensity priming stimulus [23,47].

### 2.5. Statistical Analysis

All quantitative data were processed and analyzed using the Statistical Package for the Social Sciences (SPSS), version 26.0 (IBM Corp., Armonk, NY, USA). Initially, a comprehensive descriptive synthesis was performed for all primary and secondary outcome measures. To ensure the validity of the subsequent parametric inferential models, the Shapiro–Wilk test was utilized to rigorously evaluate the normality of the data distribution within each investigative cohort independently.

To confirm the efficacy of the randomization process and establish initial stochastic equivalence, baseline inter-group comparability was interrogated. This was achieved through the application of independent-samples *t*-tests for continuous anthropometric and functional variables, while the Pearson Chi-square ($\chi^{2}$) test was utilized to assess the homogeneity of categorical demographic data, such as sex distribution and the side of the neurological insult. This rigorous preliminary screening ensures that the groups were statistically matched prior to the experimental intervention, thereby safeguarding the study against baseline selection bias.

The prime analysis employed one-way MANCOVA to compare post-intervention upper limb scores between the CT priming and standard rehabilitation groups, with the respective baseline score for each outcome entered as a covariate. This approach, modelling raw post-intervention scores as outcomes with baseline as a covariate, rather than analyzing change scores with baseline simultaneously included as a covariate, was adopted to avoid the statistical redundancy of double-adjustment and to maximize precision of estimation, in line with current methodological recommendations. Prior to conducting the MANCOVA, the following assumptions were systematically verified: multivariate normality (evaluated via Shapiro–Wilk tests on each outcome per group), homogeneity of error variances (Levene’s test), equality of covariance matrices (Box’s M test), and homogeneity of regression slopes (Group × Covariate interaction terms).

A significant overall multivariate group effect (*p* < 0.05) was the pre-specified criterion for proceeding to follow-up univariate ANCOVAs for each individual outcome, each retaining its respective baseline score as a covariate. A Bonferroni correction was applied to control the family-wise error rate across three outcomes, yielding an adjusted significance threshold of α = 0.017 per univariate test. Effect sizes were calculated as partial η^2^ for all ANCOVA models and as Hedges’s g with 95% confidence intervals for between-group comparisons, where Hedges’s g was preferred over Cohen’s d owing to its bias correction for small samples. Within-group pre-to-post improvements were assessed as secondary, descriptive analyses using paired *t*-tests, with within-group effect sizes expressed as Hedges’s g_z. These within-group comparisons were interpreted descriptively and not used to draw inferential conclusions regarding between-group efficacy, given that differential within-group significance patterns do not constitute evidence of a between-group difference. The overall significance threshold was set at *p* < 0.05 unless otherwise adjusted for multiple comparisons.

## 3. Results

### 3.1. Participant Flow and Randomization

**Figure 2:**
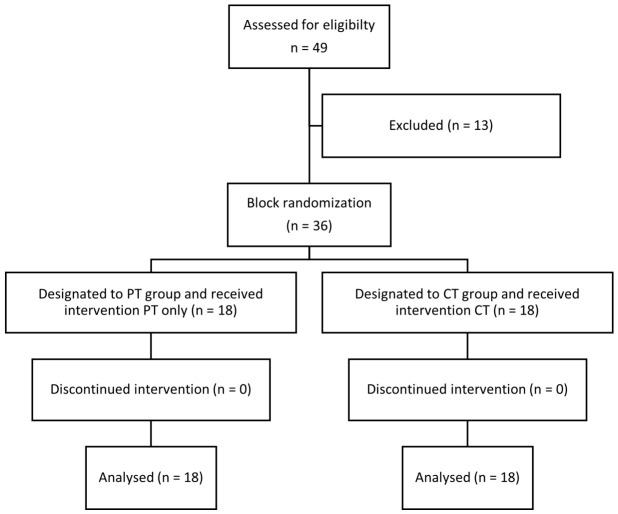
CONSORT flow diagram.

A comprehensive flowchart (Figure 2) illustrates the participants’ flow and retention through the study phases. A total of forty-nine children with unilateral cerebral palsy (UCP) were assessed for eligibility. Of these, thirteen participants were excluded—nine did not meet the inclusion criteria, and four declined to participate. The remaining thirty-six participants were randomly allocated using block randomization into two groups: the physical therapy (PT) only group (n = 18) and the combined PT and cross training (CT) group (n = 18). All participants received their allocated intervention, and no participants discontinued treatment due to excessive absence (defined as missing more than two consecutive sessions). Following the intervention period, all thirty-six participants were included in the final analysis, ensuring complete data for both groups.

### 3.2. Baseline Homogeneity

A comprehensive synthesis of the initial demographic profiles and clinical characteristics for both investigative cohorts is delineated in Table 1. Statistical interrogation of the baseline data confirmed robust inter-group comparability, with no discernible divergence identified between the PT and CT groups. The two cohorts exhibited statistical parity regarding chronological age, sex distribution, and anthropometric indices (p>0.05). Furthermore, clinical homogeneity was established concerning the side of the affected limb and the severity of spasticity, as quantified by the Modified Ashworth Scale (p>0.05).

Crucially, this stochastic equivalence extended to the primary functional outcome measures prior to the commencement of the intervention. There were no statistically significant differences between groups in the pre-test scores for handgrip strength (HGS), the Peabody Developmental Motor Scales (PDMS-FM) grasping age equivalent, or the Box and Block Test (BBT) manual dexterity scores (p>0.05). The absence of baseline variance across these diverse variables ensures a rigorous experimental foundation, confirming that any subsequent functional restoration can be objectively attributed to the specific therapeutic mechanisms of the interventions rather than confounding pre-existing disparities.

### 3.3. Differential Effects of Intervention

The MANCOVA revealed a non-significant overall multivariate effect of group (Wilks’ λ = 0.775, F_3,29_ = 2.804, *p* = 0.057, partial η^2^ = 0.225), indicating that the CT priming intervention did not produce a statistically significant differential effect on the dependent variables (i.e., HGS, PDMS-FM grasping age equivalent, and BBT score), considered as a set. Nonetheless, the borderline *p*-value and the multivariate effect size—with approximately 22.5% of the variance in the combined outcomes associated with group membership—suggest a modest, albeit inconclusive, effect that warrants cautious interpretation rather than dismissal. Given the small sample size (n = 18 per group), the study was likely underpowered to detect between-group differences of this magnitude reliably.

The follow-up univariate ANCOVAs, with respective baseline scores serving as covariates and a Bonferroni-corrected significance threshold of α = 0.017 for three outcomes, revealed no statistically significant between-group differences for any individual outcome. Specifically, no significant group difference was observed for handgrip strength (F_1,33_ = 1.980, *p* = 0.169, partial η^2^ = 0.057, Hedges’s g = −0.47, 95% CI [−1.13, 0.20]), PDMS-FM grasping age equivalent (F_1,33_ = 2.657, *p* = 0.113, partial η^2^ = 0.075, Hedges’s g = −0.50, 95% CI [−1.17, 0.16]), or the Box and Blocks Test (F_1,33_ = 0.785, *p* = 0.382, partial η^2^ = 0.023, Hedges’s g = −0.28, 95% CI [−0.94, 0.37]). Effect sizes were uniformly small, ranging from partial η^2^ = 0.023 to 0.075, and all 95% confidence intervals for Hedges’s g crossed zero, corroborating the absence of a statistically significant between-group effect. Notably, the adjusted post-intervention means—representing expected group performance at the grand mean of each respective baseline covariate—were numerically higher in the standard rehabilitation group across all three outcomes, a pattern that runs contrary to the study hypothesis (Table 2).

These findings indicate that the adjunct CT priming protocol did not confer measurable additional benefit over standard rehabilitation alone on upper limb functional outcomes within this sample. Both groups demonstrated statistically significant and clinically meaningful within-group improvements across all outcomes (all *p* < 0.010; Hedges’s g_z range: 0.66–1.27), suggesting that standard rehabilitation alone was sufficient to drive meaningful functional gains over the intervention period. The convergence of non-significant between-group effects with large within-group improvements in both arms underscores that the absence of differential efficacy cannot be attributed to a failure of the intervention context; rather, the standard rehabilitation program appeared equally potent, leaving limited variance for the CT priming to explain (Table 2).

## 4. Discussion

The present randomized controlled trial explored the feasibility of an exploratory approach in pediatric neurorehabilitation: the potential for “cross-segmental” carry-over effects induced by lower limb (LL) cross-training (CT) priming on the functional status of the contralaterally affected upper limb (UL) in children with unilateral cerebral palsy (UCP). Our primary findings revealed that while the 6-week intensive LL priming protocol approached but did not cross the traditional threshold of statistical significance in the multivariate analysis (*p* = 0.057), it demonstrated a compelling upward trajectory across all primary outcome measures. This near-threshold significance, coupled with moderate-to-large within-group effect sizes (Hedges’ g), suggests that LL cross-training acts as a supportive adjunctive mechanism, potentially creating a favorable physiological environment for subsequent therapy, though the borderline statistical trend (*p* = 0.057) necessitates a cautious, hypothesis-generating interpretation.

### 4.1. The “Neural Overflow” and Interhemispheric Modulation

Because the current trial did not utilize neuroimaging or corticospinal mapping, the mechanisms discussed below were not directly assessed and are presented strictly as hypothetical frameworks. The observed trend toward improved handgrip strength and manual dexterity following LL training may be theoretically elucidated through the lens of cortical excitability and neural “spillover.” In UCP, the non-lesioned hemisphere often exerts excessive interhemispheric inhibition (IHI) on the lesioned side, further suppressing the motor output of the affected limbs. We hypothesize that intensive strengthening of the less-affected LL (governed by the non-lesioned hemisphere) modulates this IHI balance. High-intensity resistance training has been shown to induce widespread increases in corticospinal excitability that are not strictly localized to the trained muscle group. This “neural overflow” likely lowers the activation threshold for the adjacent upper limb representations within the motor homunculus, facilitating more efficient recruitment of motor units in the paretic hand during the subsequent functional training session [48].

### 4.2. Systemic Metabolic Priming: The BDNF Mechanism

Beyond localized cortical interactions, we must consider the systemic biochemical impact of high-intensity lower limb engagement. The LL contains the largest muscle groups in the human body; their recruitment against significant resistance (Borg 4–6) triggers the release of systemic neurotrophic factors, most notably Brain-Derived Neurotrophic Factor (BDNF). As a primary mediator of neuroplasticity, BDNF enhances long-term potentiation (LTP) and synaptogenesis. It is highly plausible that LL-CT priming acted as a “metabolic catalyst,” creating a fertile neural environment that enhanced the efficacy of the subsequent Goal-Directed Task Training (GDTT). In this context, LL priming is not merely a motor task but a pharmacological-like intervention that prepares the pediatric brain for accelerated skill acquisition [49,50].

### 4.3. Biomechanical Synergy: Proximal Stability for Distal Mobility

From a biomechanical standpoint, the carry-over effects may be attributed to the optimization of the postural kinematic chain. In UCP, distal upper limb dysfunction is frequently exacerbated by “postural noise” originating from poor trunk control and pelvic instability. By strengthening the lower extremity foundation and improving weight-bearing symmetry immediately prior to UL tasks, the CT priming provided a more stable biomechanical anchor. According to the “proximal stability for distal mobility” paradigm, a stable pelvic and trunk base reduces the neural load the central nervous system must dedicate to balance. This liberates attentional and neural resources, allowing for more precise selective motor control during complex grasping (PDMS-2) and manual dexterity (BBT) tasks [51,52].

### 4.4. Statistical Nuance and the “Threshold Phenomenon”

The failure to reach absolute statistical significance (*p* < 0.05) in the between-group MANOVA must be interpreted with clinical nuance. Given the inherent heterogeneity of brain lesions in UCP, the study may have been slightly underpowered to detect the distal transfer of a secondary outcome within a 6-week window. However, the partial values and the significant within-group gains suggest a “Threshold Phenomenon.” Since both groups received an active, “Gold Standard” UL intervention, the numerical superiority of the CT group indicates that LL priming added a tangible clinical value beyond standard therapy alone. It is likely that the 6-week duration represents the “minimum effective dose,” and a more prolonged intervention might push these physiological trends into the realm of robust statistical significance.

### 4.5. Limitations

Despite the clinical relevance and the rigorous randomized design, several limitations must be addressed. First, the relatively modest sample size (n = 36) may have restricted the detection of subtle cross-segmental transfer effects. While our priori power analysis was met, the result suggests that a larger, multi-center cohort is necessary to definitively confirm these systemic trends. This “pilot-scale” sample, although typical for intensive pediatric trials, warrants caution in the broad generalization of the findings.

Secondly, while the study hypothesized a neurophysiological “spillover” effect, the absence of direct brain-mapping modalities such as functional Magnetic Resonance Imaging (fMRI) or Transcranial Magnetic Stimulation (TMS) represents a methodological constraint. Our interpretations regarding “metabolic priming” and “IHI modulation” remain highly plausible clinical hypotheses; however, the exact biological signature of the lower-to-upper limb transfer remains to be elucidated via concurrent neuroimaging data.

Furthermore, the 6-week duration may have been insufficient to consolidate these functional gains into permanent motor maps. In permanent neurological conditions like UCP, a 10–12-week training window might be required to ensure long-term retention. Additionally, the lack of a longitudinal follow-up (e.g., 6 months post-intervention) precludes conclusions regarding the “washout effect” or the sustainability of these benefits once the priming stimulus is removed. Finally, while the assessor was blinded, the nature of physical therapy prevents the double-blinding of participants and therapists, a common challenge in rehabilitative research that may introduce subtle performance bias.

### 4.6. Clinical and Research Implications

These findings provide a powerful argument for integrated limb training within the pediatric clinical landscape. By moving away from the traditional, fragmented view of cerebral palsy, therapists can optimize treatment by sequencing LL exercises before UL tasks to leverage the “window of heightened excitability.” This systemic approach recognizes the central nervous system as a holistic, interconnected network.

Future investigations should prioritize longitudinal designs with larger cohorts and incorporate biomarker analysis (e.g., systemic BDNF levels) to definitively map the trajectory of cross-segmental neuroplasticity. Expanding this research to include different UCP subtypes or broader age brackets would further clarify the optimal “therapeutic window” for cross-training priming in the pediatric population.

## 5. Conclusions

The findings of this clinical trial suggest that incorporating high-intensity lower extremity cross-training prior to standard therapy represents a feasible, though exploratory, adjunctive strategy for children with unilateral cerebral palsy. While the experimental cohort demonstrated encouraging numerical trends in upper limb performance, the near-threshold statistical results necessitate a cautious interpretation of this distal transfer effect. Although theoretical frameworks postulate that such cross-segmental improvements may stem from augmented proximal mechanical stability or interhemispheric facilitation, these underlying neurophysiological pathways remain entirely hypothetical and were not directly quantified in the current investigation. Therefore, rather than establishing a definitive clinical mandate, these preliminary results serve to generate new hypotheses. Subsequent, adequately powered, large-scale trials—ideally incorporating direct neuro-biomarkers and brain-mapping modalities—are required to substantiate the true clinical utility and biological basis of cross-segmental priming in pediatric neurorehabilitation.

## Figures and Tables

**Table 1:** Baseline synthesis of participant demographics, clinical characteristics, and pre-intervention functional metrics across investigative groups.

	PT Group (*n* = 18)	CT Group (*n* = 18)	*p*-Value
Demographic, anthropometric, and clinical measures			
Age, year	7.09 ± 0.74	7.15 ± 0.58	0.793 ^‡^
Gender (b/g), *n* (%)	12 (66.7)/6 (33.3)	10 (55.6)/8 (44.4)	1.0 ^§^
Weight, kg	27.69 ± 2.56	27.92 ± 2.18	0.781 ^‡^
Height, m	1.2 ± 0.03	1.2 ± 0.04	0.516 ^‡^
BMI, kg/m^2^	19.09 ± 1.37	19.57 ± 2.09	0.42 ^‡^
Side affected (RT/LT), *n* (%)	7 (38.9)/11 (61.1)	6 (33.3)/12 (66.7)	0.732 ^§^
MAS level (1/1+), *n* (%)	9 (50)/9 (50)	9 (50)/9 (50)	1.0 ^§^
Baseline measures of the dependent outcomes			
HGS, kg	2.94 ± 0.41	3.03 ± 0.53	0.575 ^‡^
PDMS-FM Grasping Age Equivalent, Month	26.11 ± 3.79	25.78 ± 3.26	0.785 ^‡^
BBT, Number of Blocks	10.67 ± 2.33	10.28 ± 1.71	0.571 ^‡^

PT: Physical therapy, CT: Cross-training, BMI: Body Mass Index, b/g: boy/girl, RT: right, LT: left, MAS: modified Ashworth scale, HGS: Hand grip strength, PDMS-FM Grasping Age Equivalent: Peabody Developmental Motor Scales-Fine Motor Grasping Age Equivalent, BBT: Box and Blocks test. Categorical data are expressed as frequency (%), and numerical data are shown as mean ± standard deviation. *p* values: ^‡^ unpaired *t*-test, ^§^: Pearson’s χ^2^ test.

**Table 2:** Comparative analysis of HGS, PDMS-FM Grasping Age Equivalent, and BBT Score across and within the study groups.

	PT Group (*n* = 18)	CT Group (*n* = 18)	Between-Subjects Effect	
			*p*-Value	Partial η^2^
HGS, kg				
Pre	2.94 ± 0.41	3.03 ± 0.53	0.169 *	0.0566
Post	3.49 ± 0.88	3.89 ± 0.79		
*p*-value	0.0069 *	0.0052 *		
Hedges’s g_z (95% CI)	0.721 (0.202–1.239)	0.691 (0.177–1.206)		
PDMS-FM Grasping Age Equivalent, Month				
Pre	26.11 ± 3.79	25.78 ± 3.26	0.113 *	0.0745
Post	31.62 ± 3.66	34.28 ± 6.33		
*p*-value	0.0001 *	0.00001 *		
Hedges’s g_z (95% CI)	1.268 (0.648–1.889)	1.18 (0.578–1.781)		
BBT, Number of Blocks				
Pre	10.67 ± 2.33	10.28 ± 1.71	0.382 *	0.0232
Post	12.94 ± 2.41	13.61 ± 2.17		
*p*-value	0.0095 *	0.0001 *		
Hedges’s g_z (95% CI)	1.209 (0.601–1.816)	0.659 (0.149–1.168)		

PT: Physical therapy, CT: Cross-training, HGS: Hand grip strength, PDMS-FM Grasping Age Equivalent: Peabody Developmental Motor Scales-Fine Motor Grasping Age Equivalent, BBT: Box and Blocks test, CI: confidence interval. Data are shown as mean ± standard deviation. partial η^2^: effect size of between-subject effects, Hedges’ *g_z*: *t*-test effect size. * Significant at *p* ˂ 0.05.

## Data Availability

The underlying datasets synthesized and interrogated throughout this investigation are available from the corresponding author following a legitimate and reasonable academic inquiry.

## Abbreviations

The following abbreviations are used in this manuscript:

ANCOVA	Analysis of Covariance
BBT	Box and Block Test
BDNF	Brain-Derived Neurotrophic Factor
BMI	Body Mass Index
CI	Confidence Interval
CONSORT	Consolidated Standards of Reporting Trials
CP	Cerebral Palsy
CT	Cross-Training
DSR	Deanship of Scientific Research
fMRI	Functional Magnetic Resonance Imaging
GDTT	Goal-Directed Task Training
HGS	Handgrip Strength
ICF	International Classification of Functioning, Disability and Health
IHI	Interhemispheric Inhibition
LL	Lower Limb
LTP	Long-term Potentiation
MANCOVA	Multivariate Analysis of Covariance
MANOVA	Multivariate Analysis of Variance
MAS	Modified Ashworth Scale
NDT	Neurodevelopmental Treatment
PDMS-2	Peabody Developmental Motor Scales–Second Edition
PDMS-FM	Peabody Developmental Motor Scales–Fine Motor
PT	Physical Therapy
RCT	Randomized Controlled Trial
SD	Standard Deviation
TMS	Transcranial Magnetic Stimulation
UCP	Unilateral Cerebral Palsy
UL	Upper Limb
WAQF	KAU Endowment
